# Development and validation of a practical machine learning model to predict sepsis after liver transplantation

**DOI:** 10.1080/07853890.2023.2179104

**Published:** 2023-02-15

**Authors:** Chaojin Chen, Bingcheng Chen, Jing Yang, Xiaoyue Li, Xiaorong Peng, Yawei Feng, Rongchang Guo, Fengyuan Zou, Shaoli Zhou, Ziqing Hei

**Affiliations:** a Department of Anesthesiology, The Third Affiliated Hospital of Sun Yat-sen University, Guangzhou, People’s Republic of China; b Guangzhou AID Cloud Technology Co., LTD, Guangzhou, People’s Republic of China

**Keywords:** Postoperative sepsis, liver transplantation, machine learning, early intervention, decision-making

## Abstract

**Background:**

Postoperative sepsis is one of the main causes of mortality after liver transplantation (LT). Our study aimed to develop and validate a predictive model for postoperative sepsis within 7 d in LT recipients using machine learning (ML) technology.

**Methods:**

Data of 786 patients received LT from January 2015 to January 2020 was retrospectively extracted from the big data platform of Third Affiliated Hospital of Sun Yat-sen University. Seven ML models were developed to predict postoperative sepsis. The area under the receiver-operating curve (AUC), sensitivity, specificity, accuracy, and f1-score were evaluated as the model performances. The model with the best performance was validated in an independent dataset involving 118 adult LT cases from February 2020 to April 2021. The postoperative sepsis-associated outcomes were also explored in the study.

**Results:**

After excluding 109 patients according to the exclusion criteria, 677 patients underwent LT were finally included in the analysis. Among them, 216 (31.9%) were diagnosed with sepsis after LT, which were related to more perioperative complications, increased postoperative hospital stay and mortality after LT (all *p* < .05). Our results revealed that a larger volume of red blood cell infusion, ascitic removal, blood loss and gastric drainage, less volume of crystalloid infusion and urine, longer anesthesia time, higher level of preoperative TBIL were the top 8 important variables contributing to the prediction of post-LT sepsis. The Random Forest Classifier (RF) model showed the best overall performance to predict sepsis after LT among the seven ML models developed in the study, with an AUC of 0.731, an accuracy of 71.6%, the sensitivity of 62.1%, and specificity of 76.1% in the internal validation set, and a comparable AUC of 0.755 in the external validation set.

**Conclusions:**

Our study enrolled eight pre- and intra-operative variables to develop an RF-based predictive model of post-LT sepsis to assist clinical decision-making procedure.

## Key Messages

- Postoperative sepsis is one of the main causes of mortality after liver transplantation (LT).
- Our results revealed that a larger volume of red blood cell infusion, ascitic removal, blood loss and gastric drainage, less volume of crystalloid infusion and urine, longer anesthesia time, higher level of preoperative TBIL were the top 8 important variables contributing to the prediction of post-LT sepsis.
- The Random Forest Classifier (RF) model showed the best overall performance to predict sepsis after LT in our study, which could assist in the clinical decision-making procedure.

## Introduction

Liver transplantation (LT) is currently recognized as the only effective treatment of end-stage liver disease. Although the survival rate and long-term prognosis after LT have been significantly improved in recent years due to the progress of surgical techniques, anesthetic management, immunosuppressive technology and intensive care unit (ICU) management, the LT recipients still suffered from various postoperative complications, among which postoperative sepsis was one of the most severe complications and often led to septic shock, multiple organ dysfunction syndrome (MODS) and increased postoperative mortality [1,2]. It was reported that the incidence of postoperative sepsis after LT was as high as 50–80%, and sepsis-related deaths ranged from 50% to 90% of all postoperative mortalities [3–5]. Moreover, it was reported that each 1 h delay in the treatment of sepsis would increase mortality by 7.6% [6]. Thus, a reliable model for the prediction of postoperative sepsis is critically needed to tailor preventive interventions and treatments for LT recipients.

Consequently, several predictive systems have been developed to date, including the model for end-stage liver disease (MELD), the acute physiology and chronic health evaluation (Apache-II), and the sequential organ failure assessment (SOFA) [7,8]. However, it has been noted that the accuracy and specificity of these scoring systems are unsatisfactory, especially their inability to early predict sepsis [9,10]. A meta-analysis of 42,623 patients from seven studies for predicting hospital-acquired sepsis has found that the machine learning (ML) approach had a better performance than the existing scoring systems for predicting sepsis [11].

In recent years, ML technology has been widely used in the field of intelligent medicine, which is of great practical and social significance in clinical decision-making, clinical diagnosis, and accurate medical treatment [12,13]. ML-based models have been shown to be highly accurate for predicting medical outcomes and identifying high-risk patients by taking advantage of the vast array of variables already available in the electronic patient record (EPR) [14,15]. We recently used ML to develop novel predicting models for post-LT complications, including acute kidney injury and pneumonia [16,17]. Meanwhile, ML has also been applied to establish a model to predict postoperative sepsis [18] and the outcome of death within 30 d after the operation [19]. Rishikesan Kamaleswaran recently used ML to identify ‘physiomarkers’ in continuous minute-by-minute physiologic data streams to predict the onset of sepsis after LT in postoperative ICU [20]. However, there has been no ML-based predictive model for post-LT sepsis, which might be helpful for perioperative decision-making in LT patients.

In our study, we retrospectively analyzed the perioperative data of patients receiving LT during a 6-year period from 2015 to 2021 in our hospital, aiming at establishing a ML model to predict sepsis within 7 postoperative days after LT. The findings through ML modeling may help anesthesiologists and clinicians to identify the patients at higher risk of post-LT sepsis, and apply the early intervention to reduce postoperative mortality.

## Methods

### Study subjects

The study protocol was approved by the Ethics Committee of the Third Affiliated Hospital of Sun Yat-sen University on 14 May 2021 (No. [2019]02-609-02). The requirements for informed consent and clinical trial registration were waived by the ethics committee. This study adhered to the applicable TRIPOD guidelines.

We retrospectively reviewed the LT records on the big data platform of the Third Affiliated Hospital of Sun Yat-sen University (Guangzhou, Guangdong, China) as we earlier reported [17], and the data of patients who received allogeneic LT during a 6-year period from January 2015 to April 2021 were evaluated for their eligibility during patient recruitment. All the LT recipients were registered in the China Organ Transplant Response Systems (www.cot.org.cn). The inclusion criteria were used: (1) age ≥ 18 years old; (2) allograft liver transplantation. The patients with the following conditions were excluded from this study: (1) combined liver and kidney transplantation; (2) other operations were performed at the same time; (3) incomplete medical records; (4) combined with preoperative sepsis. A total of 677 patients were enrolled, and randomly split into a training set with 70% samples (*n* = 473) and an internal validation set with the remaining 30% samples (*n* = 204), with each categorized into the following two subgroups: presence or absence of postoperative sepsis. Patients from February 2020 to April 2021 (*n* = 118) were enrolled as the external validation set.

### Primary outcome

The primary outcome of our study was defined as a machine learning model for predicting sepsis within 7 d after LT. Postoperative sepsis was diagnosed according to the diagnostic criteria of sepsis 3.0 published in the Journal of the American Medical Association in 2016 [21]. Specifically, the daily SOFA score of each patient was collected and checked manually through the electronic medical record (EMR) system. SOFA score on the day of operation was set as the baseline SOFA score. The organ dysfunction could be identified as an acute change in total SOFA score ≥ 2 points consequent to the infection.

### Variable selection

Combined with the summary of previous literature and the actual situation of our hospital, a total of 59 features were collected through the perioperative specialist database system of the electronic medical record of our hospital: demographics, preoperative data including comorbidities, etiology, complications and laboratory values, intraoperative data including incidents, medication, fluid and transfusion (Table S1). The diagnostic criteria of the postoperative complications were shown in Table S2.

As both the multicollinear variables and confounding variables would affect the model fitting performance, we implemented the least absolute shrinkage and selection operator (LASSO) regression approach to select the features with non-zero coefficients after LASSO regression [22], so as to prevent over-fitting of the model and enhance its clinical applicability. Meanwhile, the bootstrap method was used with the LASSO method to sample 1000 different test sets and deal with the instability and sensitivity of LASSO regression to sampling variability.

### Development and validation of the ML model

We compared seven predictive modeling approaches: Logistic Regression (LR), Support Vector Machine (SVM), Random Forest Classifier (RF), Gradient Boosting Machine (GBM), Adaptive Boosting Classifier (ADA), Gaussian Naive Bayes (GNB), and Multi-layer Perceptron (MLP). All the above models were established *via* the Scikit-learn package (https://github.com/scikit-learn/scikit-learn). The completed data was separated into a 70% training set and a 30% validation set. The Bootstrap method was then applied to get a 95% confidence interval (CI) of evaluation metrics for each model. We used the area under the receiver operating characteristic curve (AUC), sensitivity, specificity and F1-score to evaluate the model performance. The SHapley Additive exPlanations (SHAP) method was applied to evaluate feature importance and explain the predictions made by ML algorithms. Meanwhile, as the SOFA score has been used to predict the occurrence of sepsis in the ICU, we also compared the performance of our ML model to the SOFA score in the study.

### Statistical analysis

Analyses were implemented under an anaconda base environment (https://www.anaconda.com) with python 3.7. The dependent package included: scikit-learn 0.22, numpy 1.17.0, pandas 1.2.3. We used independent sample *t*-test to compare normally distributed data, as for non-normal distribution data, we used Mann–Whitney *U*-test in univariate analyses. Categorical variables were tested by Chi-square test or Fisher’s exact test when cell counts less than five. Kaplan–Meier methods were applied to estimate the long-term survival rates.

Only categorical variables had missing values and the missing proportion was less than 10%. Mode imputation was for the categorical variables. Continuous variables in both the training and validation set were normalized base on the mean and standard deviation of the training set, while categorical variables were dummy coded. All models would be developed in the same 70% training set and validated in 30% validation set. For further application, we built an online risk calculator to help clinical decision-making of postoperative sepsis.

## Results

**Figure 1.**
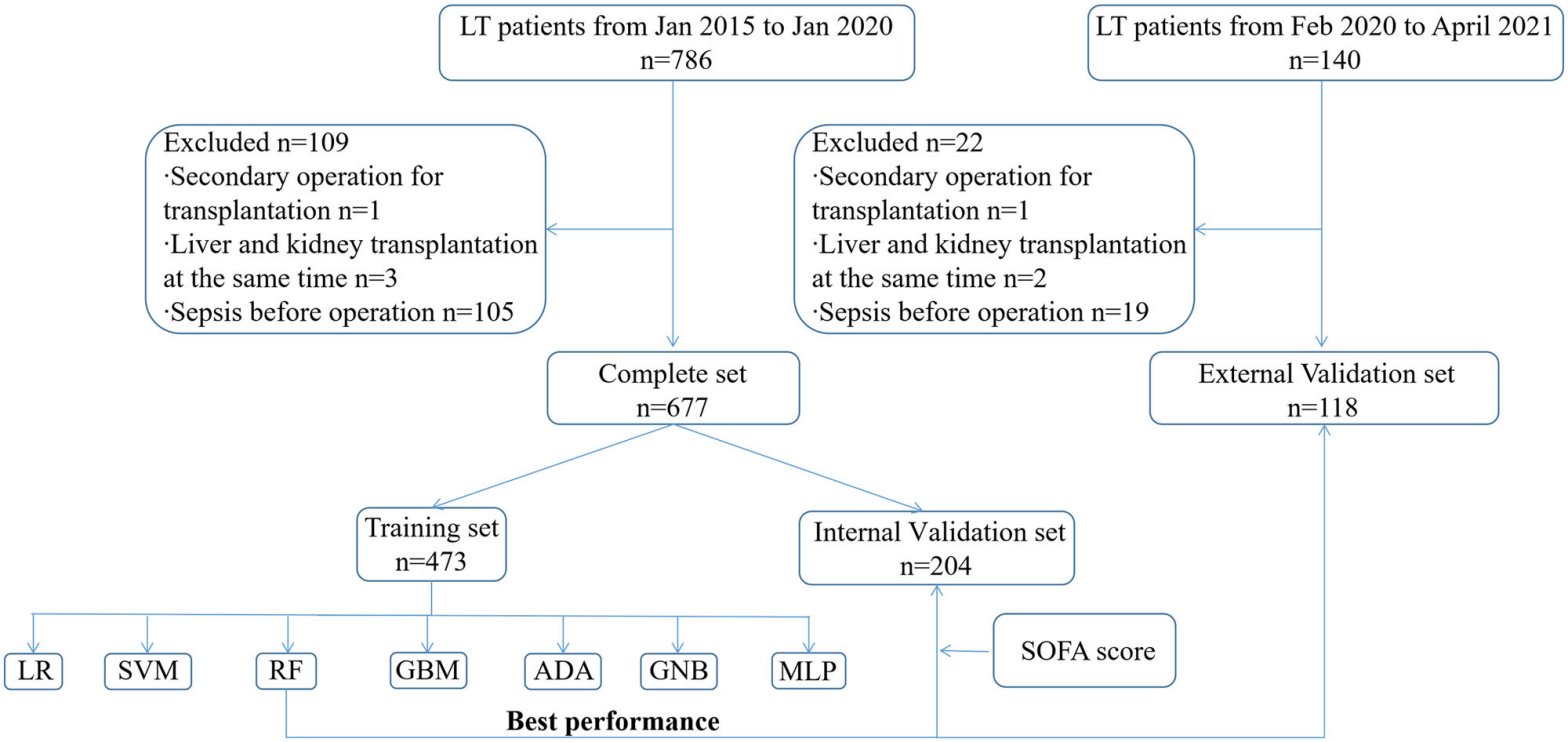
Flow chart of patient enrollment. LR: logistic regression; SVM: Support Vector Machine; RF: Random Forest; GBM: Gradient Boosting Machine; ADA: Adaptive Boosting Classifier, KNN: K Nearest Neighbors Classifier; DT: Decision Tree Classifier.

786 patients underwent LT in our hospital were assessed in our study. We excluded one patient for re-transplantation for graft failure, 3 patients for simultaneous liver and kidney transplantation and 105 patients for sepsis before the operation. 677 patients were finally enrolled in our study. The incidence of postoperative sepsis was 31.9% in our study. The flow chart of patient enrollment was showed in Figure 1.

### Preoperative characteristics of patients with or without sepsis

The preoperative characteristics of the patients with or without sepsis after LT were presented in Table 1. The patients with postoperative sepsis showed significant lower levels of hematocrit (HCT), platelets (PLT), hemoglobin (HGB) and albumin (ALB), as well as higher levels of white blood cell count (WBC), aspartate amino transferase (AST), total bilirubin (TBIL), indirect bilirubin (IBIL), direct bilirubin (DBILI), blood urea nitrogen (BUN), prothrombin time (PT), activated partial thromboplastin time (APTT) and international normalized ratio (INR) (all *p* < .05) than those without sepsis. Moreover, the patients with postoperative sepsis had higher MELD score and Child-Pugh score (both *p* < .001), and preoperative ICU stay and incidence of preoperative tracheal intubation were found to have significant differences between patients with or without postoperative sepsis (both *p* < .001). While gender, age, height, weight, BMI and ASA classification were found to have no significant difference between patients with or without sepsis.

**Table 1.** Preoperative characteristics of the patients.

Variables	Patients without sepsis (*n* = 461)	Patients with sepsis (*n* = 216)	*p*-Value
Gender			.403
Male	401.00 (86.99%)	182.00 (84.26%)	
Female	60 .00 (13.01%)	34.00 (15.74%)	
Age (y)	48.80 ± 10.40	49.60 ± 10.50	.541
Height (cm)	167.70 ± 6.50	167.0 0 ± 6.50	.320
Weight (kg)	64.60 ± 10.60	65.00 ± 11.50	.834
Body mass index	23.00 ± 3.40	22.80 ± 3.10	.675
ASA classification			.536
2	52.00 (11.28%)	21.00 (9.73%)	
3	409.00 (88.72%)	195.00 (90.27%)	
Comorbidities			
Heart failure (*n*)	6.00 (1.30%)	4.00 (1.90%)	.832
Myocardial infarction (*n*)	16.00 (3.47%)	22.00 (10.19%)	**.001**
Diabetes mellitus (*n*)	54.00 (0.16)	30.00 (0.12)	.194
Hepatic encephalopathy (*n*)	133.00 (28.9%)	76.00 (35.19%)	.116
Acute liver failure (*n*)	135.00 (29.28%)	104.00 (48.15%)	**<.001**
Subacute liver failure (*n*)	220.00 (47.72%)	151.00 (69.91%)	**<.001**
Hypokalemia (*n*)	97.00 (21.04%)	49.00 (22.69%)	.701
Etiology for liver transplantation			
Hepatitis B (*n*)	372.00 (80.5%)	165.00 (76.4%)	.217
Hepatitis C (*n*)	14.00 (3.00%)	6.00 (2.80%)	.856
Hepatic malignancy (*n*)	233.00 (50.40%)	73.00 (33.80%)	**<.001**
Biliary cirrhosis (*n*)	13.00 (2.80%)	6.00 (2.80%)	.979
Alcoholic cirrhosis (*n*)	38.00 (8.20%)	15.00 (7.00%)	.563
Laboratory results			
HCT	0.31 (0.07)	0.29 (0.07)	**<.001**
Platelets (10**^9^**/L)	100.56 (72.09)	86.55 (61.73)	**.004**
WBC (10**^9^**/L)	6.06 (4.58)	7.77 (5.12)	**<.001**
Lymphocyte (10^9^/L)	1.03 (0.57)	1.05 (0.6)	.821
HGB (g/L)	105.86 (24.66)	97.46 (23.2)	**<.001**
ABO			.866
A	124.00 (26.90%)	59.00 (27.32%)	
B	116.00 (25.16%)	60.00 (27.78%)	
O	187.00 (40.56%)	83.00 (38.43%)	
AB	34.00 (7.38%)	14.00 (6.48%)	
ALT (U/L)	83.85 (171.68)	106.25 (225.95)	.228
AST (U/L)	111.23 (268.73)	113.89 (144.16)	**.002**
GGT (g/L)	110.75 (165.48)	85.85 (120.00)	**<.001**
TBIL (μmol/L)	195.68 (214.05)	311.42 (248.00)	**<.001**
IBIL (μmol/L)	68.28 (75.38)	116.96 (102.47)	**<.001**
DBILI (μmol/L)	127.54 (149.05)	194.47 (165.96)	**<.001**
ALB (g/L)	35.97 (4.93)	34.97 (4.11)	**.028**
CHOL (mmol/L)	3.33 (1.37)	3.03 (1.57)	**.001**
Last SCr (μmol/L)	79.58 (37.31)	95.92 (72.61)	.090
BUN (mmol/L)	5.67 (3.61)	7.75 (7.37)	**.004**
PT (s)	22.84 (10.75)	27.12 (13.5)	**<.001**
APTT (s)	51.21 (15.03)	57.68 (22.14)	**<.001**
INR	2.04 (1.18)	2.53 (1.44)	**<.001**
Serum potassium (mmol/L)	3.81 (0.45)	3.86 (0.49)	**.030**
Serum sodium (mmol/L)	138.68 (4.57)	138.48 (5.21)	.261
Serum calcium (mmol/L)	2.30 (0.17)	2.33 (0.23)	.529
Complications and treatments			
MELD score	18.20 ± 10.80	24.70 ± 11.50	**<.001**
Child-Pugh score	8.90 (2.40)	9.70 (2.10)	**<.001**
Preoperative ICU stay (*n*)	205.00 (44.47%)	143.00 (66.20%)	**<.001**
Renal replacement therapy	51.00 (15%)	32 .00 (13%)	.468
Preoperative tracheal intubation	182.00 (39.48%)	114.00 (52.78%)	**.002**

Note: Data were expressed as frequency (proportion) or mean (SD). Bold data indicates significance at *p* < .05. WBC: white blood cell; ALT: alanine transaminase; AST: aspartate amino transferase; TBIL: total bilirubin; IBIL: indirect bilirubin; ALB: albumin; BUN: blood urea nitrogen; PT: prothrombin time; APTT: activated partial thromboplastin time; INR: international normalized ratio; Hypokalemia was defined as the serum concentration of potassium level is below 3.5 mmol/L. Bold data indicates significance at *p* < .05.

### Intraoperative characteristics of patients with or without sepsis

The intraoperative factors were compared between the patients without or with postoperative sepsis (Table 2). The patients with postoperative sepsis had longer anesthesia time, more red blood cell (RBC), cryoprecipitate and sodium bicarbonate transfusion, and less crystalloid transfusion and urine output (all *p* < .05). Meanwhile, the volume of blood loss, ascites removal and gastric drainage were significantly higher in the sepsis group than the non-sepsis group (Table 2).

**Table 2.** Comparison of intraoperative factors between two groups.

Variables	Patients without sepsis (*n* = 461)	Patients with sepsis (*n* = 216)	*p*-Value
Cold ischemic time (h)	6.03 (1.19)	6.14 (1.19)	.091
Anesthesia time (min)	508.79 (113.34)	536.52 (170.99)	**<.001**
Intraoperative fluid management and transfusion			
Crystalloid (mL)	2680.13 (1763.62)	2230.26 (2208.56)	**.001**
Colloid (mL)	109.38 (306.45)	117.42 (550.95)	.379
RBC transfusion (mL)	1097.13 (884.96)	1818.6 (1363.29)	**<.001**
Plasma transfusion (mL)	1780.05 (1260.09)	1846.3 (1860.96)	.714
Cryoprecipitate transfusion (U)	37.59 (70.41)	46.14 (89.63)	**<.001**
Sodium bicarbonate transfusion (mL)	96.18 (165.32)	182.52 (701.74)	**.011**
Albumin (mL)	282.73 (413.77)	321.88 (607.9)	.665
Total volume of infusion (mL)	5308.54 (3823.7)	5259.84 (5692.81)	.533
Blood loss (mL)	1510.9 (1342.5)	2285.71 (2197.76)	**<.001**
Urine output (mL)	1718.74 (979.52)	1538.69 (1055.11)	**.010**
Ascites removal (mL)	732.66 (1776.81)	1097.73 (1958.25)	**.023**
Gastric drainage (mL)	37.19 (97.7)	80.10 (297.54)	**.005**
Total volume of output (mL)	3211.39 (2703.44)	3029.81 (3314.36)	.074

Note: Continuous variables were presented with mean along with standard deviation (SD), or median (interquartile range). IV: intravenous injection.

data indicates significance at *p* < .05.

### Effect of sepsis on patient outcomes and prognosis

Compared with the patients without postoperative sepsis, the patients with postoperative sepsis were combined with more perioperative complications, including higher incidence of abdominal infection (4.6% *vs.* 0%, *p* < .001), bile tract infection (5.1% *vs.* 2.2%, *p* = .040; Table 3), pneumonia (56.9% *vs.* 43.3%, *p* = .001; Table 3), kidney failure (7.4% *vs.* 0.6%, *p* < .001) and hepatorenal syndrome (6.5% *vs.*1.1%, *p* < .001; Table 3). Moreover, patients with postoperative sepsis also had longer postoperative ICU stay [3 (2–4) *vs.* 5(4–8) d, *p* < .001; Table 3], longer postoperative hospital stay [20 (16–26) *vs.* 29 (21–39) d, *p* < .001; Table 3], and more hospitalization cost [0.27 (0.23–0.32) *vs.* 0.38 (0.31–0.51) million-yuan, *p* < .001; Table 3].

**Table 3.** Comparison of complications and prognosis between two groups.

Variables	Patients without sepsis (*n* = 461)	Patients with sepsis (*n* = 216)	*p*-Value
Cardiac-related complications	21 (4.5%)	12 (5.6%)	.569
Abdominal infection	0 (0%)	10 (4.6%)	**<.001**
Bile tract infection	10 (2.2%)	11 (5.1%)	**.040**
Pneumonia	200 (43.3%)	123 (56.9%)	**.001**
Kidney failure	3 (0.7%)	16 (7.4%)	**<.001**
Hepatorenal syndrome	5 (1.1%)	14 (6.5%)	**<.001**
Postoperative ICU stay (d)	3 (2–4)	5 (4–8)	**<.001**
Postoperative length of stay (d)	20 (16–26)	29 (21–39)	**<.001**
Hospitalization cost (million)	0.27 (0.23–0.32)	0.38 (0.31–0.51)	**<.001**
Survival rate at 30 d after LT	456 (98.9%)	194 (90.2%)	**<.001**
Survival rate at 90 d after LT	452 (98.0%)	176 (81.9%)	**<.001**

Note: Data were expressed as frequency (proportion) or median (interquartile range). LT: liver transplantation.

Bold data indicates significance at *p <* .05.

Furthermore, as shown in Table 3, we found that the patients with postoperative sepsis had significant lower survival rate at 30 d (90.2% *vs.* 98.9%, *p* < .001) and 90 d (81.9% *vs.* 98.0%, *p* < .001).

### Feature selection using LASSO regression

**Figure 2.**
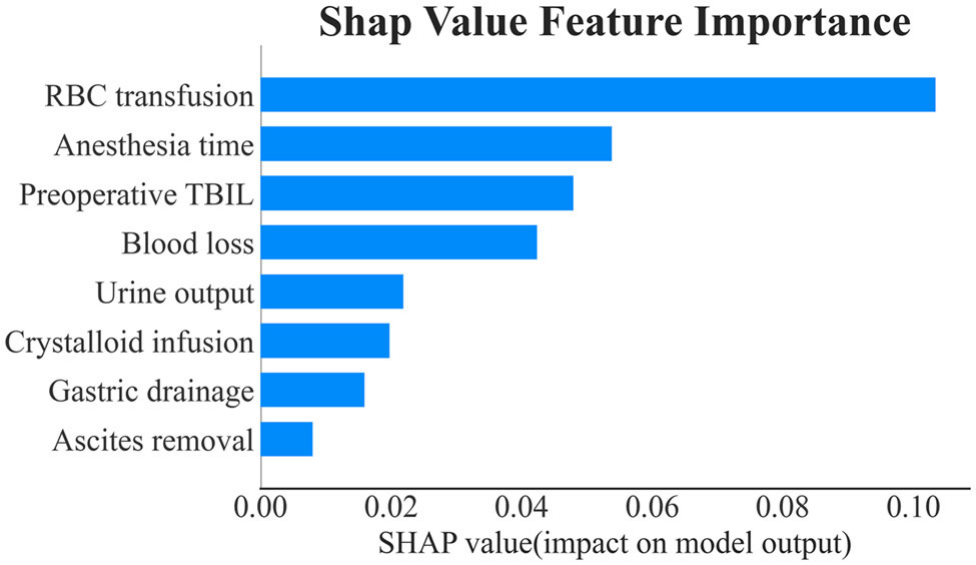
Feature importance ranking of the selected 8 features.

Finally, 59 features were reduced to 8 potential predictors on the basis of 677 patients in the primary cohort, and these eight features, chosen to build machine learning models in the model building part, included RBC transfusion, anesthesia time, preoperative TBIL, blood loss, urine output, crystalloid infusion, gastric drainage, ascites removal (Figure 2). Further, feature importance plot was created to rank the levels of importance. As a result, RBC transfusion, anesthesia time and preoperative TBIL were ranked first, second, and third, respectively (Figure 2).

### Performance assessment of the ML models to predict sepsis after LT

**Figure 3.**
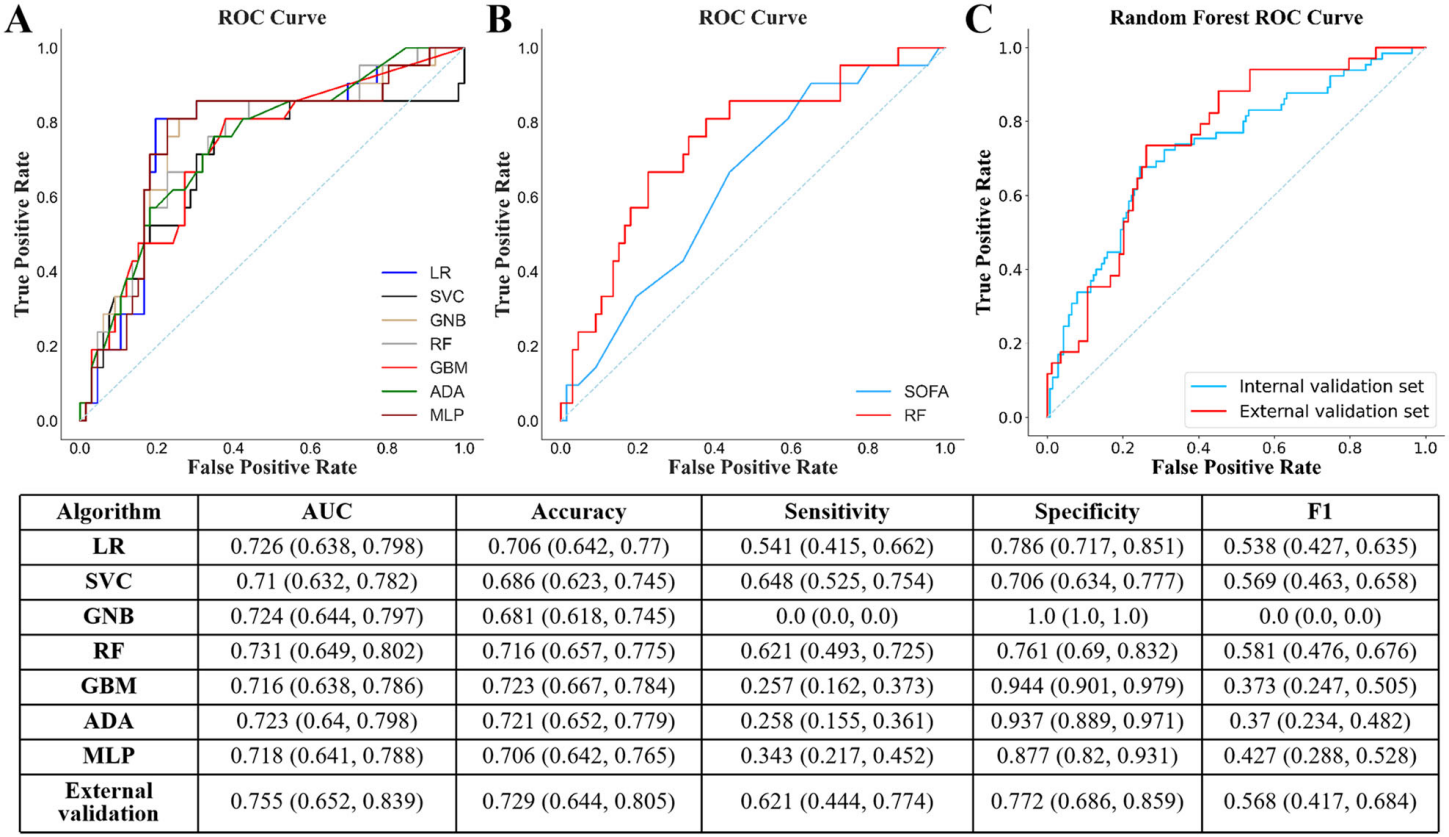
ROC curve for prediction of postoperative sepsis. (A) Performance of all predicting models in the internal validation set. (B) ROC curve of the RF model and SOFA score in the validation set. (C) Performance of RF model in the internal validation set and on the external validation set.

Among the seven predictive modeling approaches in the study, RF model achieved the greatest AUC (0.731, CI 0.649–0.802), the highest F1-score (0.581, CI 0.476–0.676), and relatively balanced sensitivity (0.621, CI 0.493–0.725) and specificity (0.761, CI 0.69–0.832) (Figure 3). Thus, we eventually chose RF model for further analysis and application (Figure 3(A)).

Since the SOFA score has been reported to predict postoperative sepsis in ICU patients, we validated and compared the performance of this score and our RF-based models in the validation set (Figure 3(B)). It turned out that the SOFA score presented in our validation set had a lower AUC (0.637, CI 0.551–0.692) than the RF model (0.745, CI 0.645–0.824) in the validation set.

### Temporal external validation

The external validation set also consisted of a majority of male (90.68%) with a mean age of 47.3 years old (Table S3). On one hand, the anesthesia time and volume of urine output were significantly lower in the external validation set compared to that of the development set (both *p* < .05). On the other hand, the volume of crystalloid infusion and ascites removal were higher in the external validation set. In this temporal validation set, the incidence of sepsis was 28.81%, and the RF model achieved a comparable AUC (0.755, CI 0.652–0.839) to that of the internal validation set (Figure 3(C)).

### Predictive online risk calculator

**Figure 4.**
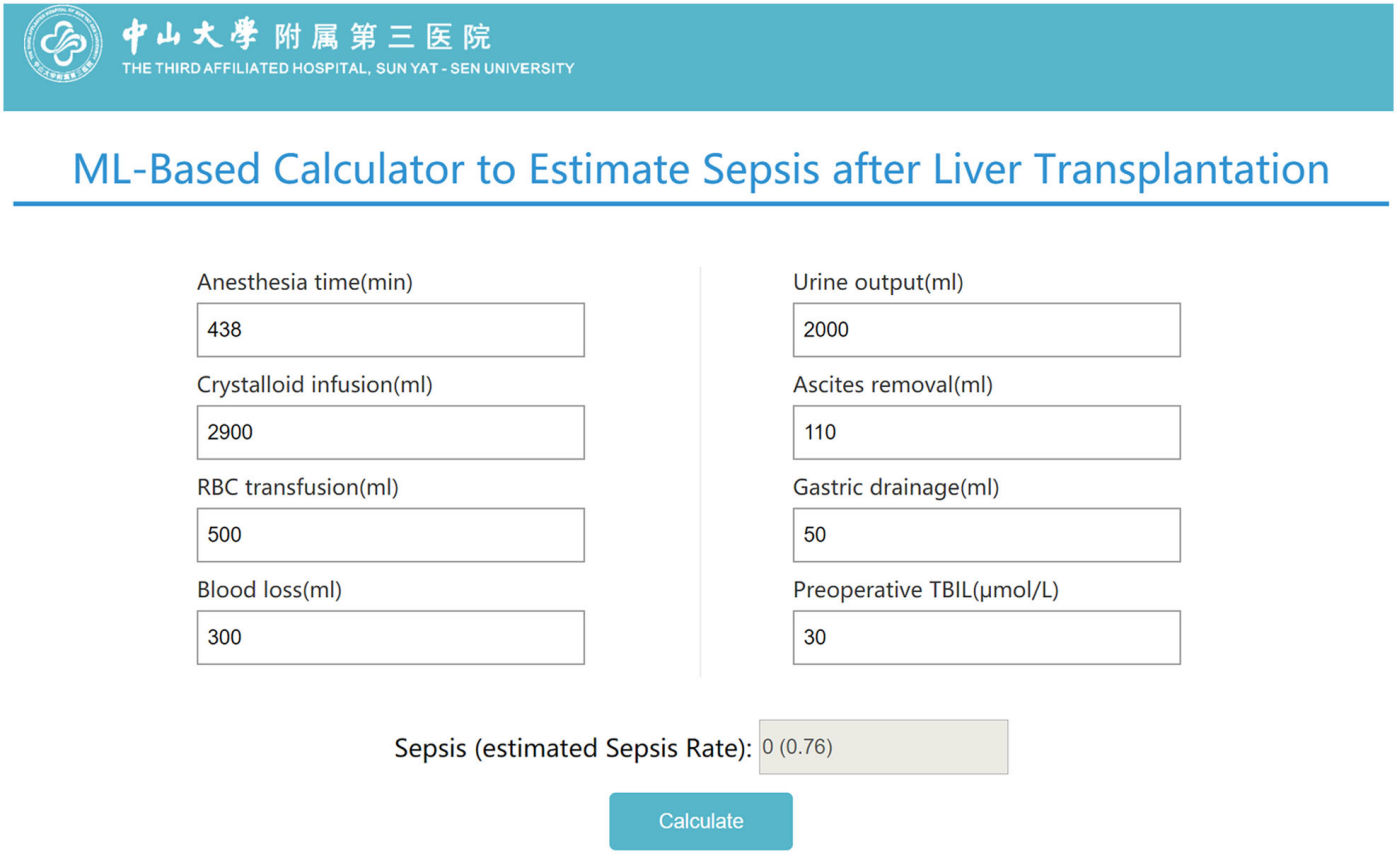
Online calculator of risk for sepsis after LT. A demo prediction of patient No.10 by online ML-based predictor of post-LT Sepsis. ‘1’ represents a positive result, and ‘0’ represents a negative result. The value in parentheses is the occurrence probability of post-LT sepsis. For instance, the prediction output for patient No. 10 was ‘0’ with a probability of 76%, that is, the probability of this patient developing post-LT sepsis was only 24%. The browse-based tool can be visited at http://wb.aidcloud.cn/zssy/sepsis_web.html.

As the eight variables enrolled in our model could be easily obtained in clinical practice to calculate the risk of sepsis after LT conveniently, we also developed an online risk calculator to make the RF model accessible to anesthesiologists and peers around the world. As shown in Figure 4, ‘1’ represents a positive result, and ‘0’ represents a negative result. The value in parentheses is the occurrence probability of post-LT sepsis. For instance, the prediction output of patient No. 10 was ‘0’ with a probability of 76%, that is, the probability of this patient developing post-LT sepsis was only 24%. The online risk calculator to calculate the risk of sepsis of LT can be accessed at http://wb.aidcloud.cn/zssy/sepsis_web.html.

## Discussion

In this study, we evaluated the ability of seven machine learning algorithms to predict postoperative sepsis in LT patients and generated the following major practical findings: (1) The incidence of postoperative sepsis was up to 31.9% in patients after LT, and the occurrence of sepsis was significantly associated with more perioperative complications, prolonged postoperative ICU stay and hospital stay, more hospitalization cost and increased mortality at 30 d and 90 d after LT; (2) A total of 8 factors were identified to be significantly correlated with postoperative sepsis after LT, including RBC transfusion, anesthesia time, preoperative TBIL, blood loss, urine output, crystalloid infusion, gastric drainage, ascites removal; (3) The random forest classifier model exhibited the best overall performance to predict sepsis after LT among the seven developed ML models, with an AUC of 0.731, an accuracy of 71.6%, a sensitivity of 62.1%, and a specificity of 76.1%.

Sepsis is a common and major health crisis in hospitals globally [23]. We reported the high incidence of sepsis and related adverse prognosis after LT, which verified the significance of early prevention and treatment of sepsis after LT in increasing individual survival time, improving quality of life and reducing the burden of the health care system. Consistently, it was strongly recommended to perform sepsis screening and accurate prediction for acutely ill, high-risk patients [24], including LT patients who are at elevated risk of developing sepsis [25].

To date, an ML-based predictive model for postoperative sepsis after LT has been developed to assist clinicians in postoperative decision-making and prevention in clinical practice [20]. However, the model was established only using continuous minute-by-minute physiologic data streams in postoperative ICU to predict the onset of sepsis after LT and was unable to assist intraoperative decision-making in LT patients. As we known, risk factors of LT-related sepsis were reported to exist in the whole perioperative period including pre-, intra- and post-operation [26–28]. Considering that the clinical application of postoperative factors in predicting diseases had certain limitations due to its time lag, we collected 59 variables from preoperative and intraoperative data in this study with the aim to predict LT-related sepsis at an earlier stage and enable the anesthesiologists to apply early intervention during operations. During the operation, all variables involved in the RF model could be collected in real-time, it, therefore, allowed us to perform the prediction model during the LT surgery in real-time with the updated data. Actually, we hope to calculate the estimated incidence of sepsis using the model during the whole procedure, and if the prediction showed a positive result of postoperative sepsis, the anesthesiologists could combine their clinical experience and pay more attention to the hemodynamic stability and appropriately start fluid resuscitation. After surgery, it would be an early warning sign of sepsis and close attention would be given by the ICU physicians to these patients. For instance, blood cultures were recommended to be drawn and diagnosis could be made early to choose appropriate treatment strategies.

The LASSO method, suitable for the regression of high-dimensional data, was used to select the most useful predictive features from the primary data set [22]. On one hand, it could reduce variables and prevent overfitting of the model; on the other hand, it was also more convenient to obtain data and reduce the cost of obtaining data in practical application. With it, eight variables that widely used and routinely recorded were enrolled in our ML models, including preoperative TBIL, intraoperative RBC transfusion, anesthesia time, blood loss, urine output, crystalloid infusion, gastric drainage and ascites removal. Notably, all these variables can be explained by pathophysiology and clinical knowledge, which holds promise for clinical application in predicting sepsis for patients after LT in the future.

In our study, the RF model had the best overall performance in predicting postoperative sepsis, with the greatest AUC of 0.731, the highest F1-score of 0.581, and relatively balanced specificity and sensitivity of 76.1% and 62.1%. Consistent with an earlier study [9,10], we found the RF model had a higher performance than that of the postoperative SOFA score. The RF model refers to a classifier that uses multiple trees to train and predict samples and it has the advantage of speed for the training of large samples, with small model variance, and strong generalization ability. Although the RF model had sufficient specificity, its sensitivity in predicting postoperative sepsis in LT patients was a little weak (only 62.1%), and this may be due to the fact that the risk factors of sepsis after LT were relatively complex and the weight of the same factor to different patients with sepsis was different. In addition, the random forest algorithm is also easy to over-fit in such noisy sample sets. Nevertheless, as an ensemble ML model, RF improves its classification by using the bagging method to aggregate multiple (usually hundreds) decision trees. More specifically, since its bagging nature are more resistant to noisy samples and observations, RF is more stable than other models in predicting postoperative sepsis [29,30].

Several limitations in our study should be noticed. First, due to the retrospective design, possible collection bias, entry bias, and residual confounding may occur, and we did not include the patients with preoperative sepsis, which might be the major predictor of postoperative sepsis. Second, a low platelet count, a high level of bilirubin, the need for vasopressors and acute kidney injury lead to an increase in the SOFA score. But with the same clinical picture, postoperative graft dysfunction can look very similar, including thrombocytopenia, high bilirubin, kidney failure and the need for vasopressors. Though there are several similar indicators with SOFA score, sepsis is an infection-initiated dysregulated host response involving multiple organs. Infection symptoms and other organ dysfunction might help clinicians to differentiate. Third, based on the accuracy of the model and the percent of septic patients in the external validation data set, it missed 8% of septic patients, which made it to be used only as a decision-making aid to the clinicians, instead of being a diagnostic tool. Fourth, our study is a single center study due to the lack of data from other transplantation centers. we validated our model in a temporally independent dataset, which is considered to be a kind of controversial but acceptable external validation in the TRIPOD statement (Type2b), an intermediary between internal and external validation [31]. Furthermore, we also developed an online risk calculator to make the RF model accessible to anesthesiologists and peers around the world for external validation.

## Conclusions

The current study has established a RF-based ML model that enrolled preoperative and intraoperative variables to predict sepsis after LT, which holds promise for future clinical application to predict postoperative sepsis in LT recipients.

## Supplementary Material

Supplemental Material

## Data Availability

All data generated or analyzed during this study are included in this published article and its supplementary information files.
