# Supplementary material for: Development and validation of a practical machine learning model to predict sepsis after liver transplantation
Source: Ann Med. 2023 Feb 15;55(1):624–33. doi: 10.1080/07853890.2023.2179104 (PMC9937004; doi:10.1080/07853890.2023.2179104)
Supplement: Supplemental Material [file IANN_A_2179104_SM3231.docx]

**Supplementary File**

**Development and validation of a practical machine learning model to predict sepsis after liver transplantation**

Chen Chaojin^#1^; Chen Bingcheng^#1^; Yang Jing^#1^; Li Xiaoyue^1^; Peng Xiaorong^1^; Feng Yawei^1^; Lai Bingchang^2^; Zou Fengyuan^2^; Zhou Shaoli*^1^; Hei Ziqing*^1^

^1^Department of Anesthesiology, The Third Affiliated Hospital of Sun Yat-sen University, Guangzhou, People’s Republic of China.

^2^Guangzhou AID cloud technology co., LTD, Guangzhou, People’s Republic of China.

**Table S1. List of the 59 features enrolled in the study**

| **Categories** | **Variables** |
| --- | --- |
| Demographics (6) | Gender, Age, Height, Weight, Body Mass Index, ASA classification |
| Preoperative Variables |  |
| Comorbidities (7) | Heart failure, Myocardial infarction, Diabetes mellitus, Hepatic encephalopathy, Acute liver failure, Subacute liver failure, Hypokalemia |
| Etiology (4) | Viral hepatitis (Hepatitis B, Hepatitis C), Hepatic malignancy, Biliary cirrhosis, Alcoholic cirrhosis |
| complications (5) | MELD score, Child-Pugh score, Preoperative ICU stay, Renal replacement therapy, Preoperative tracheal intubation |
| laboratory values (22) | Hematocrit, Platelets, WBC, Lymphocyte, HGB, ABO, ALT, AST, GGT, TBIL, IBIL, DBILI, ALB, CHOL, Last SCr, BUN, PT, APTT, INR, Serum potassium, Serum sodium, Serum calcium |
| Intraoperative Variables |  |
| Incidents (2) | Cold Ischemic Time, Anesthesia Time |
| fluid and transfusion (13) | Crystalloid, Colloid, RBC transfusion, Plasma transfusion, Cryoprecipitate transfusion, Sodium bicarbonate transfusion, Albumin, Total volume of infusion, Blood loss, Urine output, Ascites removal, Gastric drainage, Total volume of output |

**Table S2. Diagnostic criteria of the postoperative complications**

| **Complications** | **Definitions** |
| --- | --- |
| Intra-abdominal Infection | Intra-abdominal infections comprise a very heterogeneous group of infectious processes that share an anatomical site of origin between the diaphragm and the pelvis, defined according to the criteria of the International Sepsis Forum (ISF)^1^. |
| Bile Tract Infection | Cholecystitis was diagnosed on the basis of clinical presentation of fever, right upper quadrant pain, and findings of ultrasonography or computed tomography. Cholangitis was diagnosed based on the following criteria: (a) presence of fever with upper quadrant pain; (b) radiological (sonographic or computed tomographic) or endoscopic evidence of biliary tract obstruction due to stones or stricture from benign or malignant origin; and (c) laboratory findings of hyperbilirubinemia and an elevated serum alkaline phosphatase level^2^. |
| Pneumonia | Postoperative pneumonia was defined on the basis of European Perioperative Clinical Outcome (EPCO) definitions^3^. |
| Kidney Failure | Kidney Failure was defined as the acute loss of kidney function according to an established RIFLE criterion^4^. |
| Hepatorenal Syndrome (HRS) | HRS was defined as renal dysfunction that occurs because of reduced renal perfusion, due to haemodynamic alterations in arterial circulation, as well as overactivity of the endogenous vasoactive systems^5^. |

**Reference**

1. Calandra T, Cohen J. The international sepsis forum consensus conference on definitions of infection in the intensive care unit. *Crit Care Med.* 2005;33(7):1538-1548.

2. Kawanishi K, Kinoshita J, Abe H, et al. Appendectomy as a Risk Factor for Bacteremic Biliary Tract Infection Caused by Antibiotic-Resistant Pathogens. *Biomed Res Int.* 2017;2017:3276120.

3. Chen C, Yang D, Gao S, et al. Development and performance assessment of novel machine learning models to predict pneumonia after liver transplantation. *Respir Res.* 2021;22(1):94.

4. Lopes JA, Jorge S. The RIFLE and AKIN classifications for acute kidney injury: a critical and comprehensive review. *Clin Kidney J.* 2013;6(1):8-14.

5. Angeli P, Garcia-Tsao G, Nadim MK, Parikh CR. News in pathophysiology, definition and classification of hepatorenal syndrome: A step beyond the International Club of Ascites (ICA) consensus document. *J Hepatol.* 2019;71(4):811-822.

**Table S3 Comparison of variables between the Training set and the external validation set**

| **Characteristics** | **Training set**  **(n = 473)** | **External Validation set**  **(n = 118）** | ***P*_value** |
| --- | --- | --- | --- |
| Gender |  |  | 0.43 |
| Male | 407(86.05%) | 107.00(90.68%) |  |
| Female | 66(13.95%) | 11.00(9.32%) |  |
| Age | 49.21(10.59) | 47.28 (10.66) | 0.077 |
| Anesthesia time | 522.15(141.62) | 448.17(109.45) | **0.048** |
| Crystalloid infusion | 2617.69(1829.05) | 3632.2(2044.47) | **< 0.001** |
| RBC transfusion | 1325.22(1082.83) | 1232.95(1227.44) | 0.421 |
| Blood loss | 1779.61(1754.89) | 1579.87(2090.08) | 0.288 |
| Urine output | 1663.62(977.58) | 1447.34(868.88) | **0.035** |
| Ascites removal | 828.64(852.11) | 1222.88(1127.85) | **0.047** |
| Gastric drainage | 57.61(218.07) | 74.53(162.74) | 0.43 |
| Preoperative TBIL | 236.77(226.17) | 239.96(225.95) | 0.891 |

Note: Data were expressed as mean (SD). Bold data indicates significance at *P* < 0.05.
